# Sexual dysfnction in Iranian pregnant women

**Published:** 2013-06

**Authors:** Safieh Jamali, Leili Mosalanejad

**Affiliations:** 1 *Department of Midwifery, Faculty of Nursing and Midwifery, Jahrom University of Medical Sciences, Jahrom, Iran.*; 2 *Deaprtment of Psychiatry, Jahrom University of Medical Sciences, Jahrom, Iran.*

**Keywords:** *Sexual dysfunction*, *Pregnancy*, *Female Sexual Function Index*

## Abstract

**Background:** Sexuality is an important part of women’s health, quality of life, and general well-being. There are many factors influencing the female sexual function, including psychological, physiological, couple relationship, and socio-cultural factors. Pregnancy plays an important role in the sexual function and behavior of women.

**Objective:** This study aims to evaluate the sexual function and determine the prevalence of sexual dysfunction among women during pregnancy.

**Materials and Methods:** The present cross-sectional study was conducted on 257 healthy pregnant women aging between18-40 years who had attended the antenatal clinic, Paymaneh Hospital, Jahrom, Iran between April and October 2011 Female Sexual Function Index (FSFI) questionnaire was used for assessing the sexual function

**Results:** The mean age of the participants was reported as 26.45±4.49 years. In addition, 143, 69, and 45 subjects were in their 1^st^, 2^nd^, and 3^rd^ trimesters, respectively. Comparison of the second and the third trimesters revealed a significant difference in the scores of all FSFI domains and the mean total FSFI score was reported as 19.9±22.45. Among the study subjects, 197 ones (79.1%) had sexual dysfunction (FSFI score <26.5), while only 52 (20.9%) had normal sexual function (FSFI score ≥26.5). The sexual dysfunction among pregnant women was rated as 23.4%, 30.5%, and 46.2% in the 1^st^, 2^nd^, and 3^rd^ trimesters, respectively.

**Conclusion:** The prevalence of sexual dysfunction is high during pregnancy and reaches higher levels in the third trimester. Therefore, pregnant women and their partners need counseling about physical and psychological changes in pregnancy.

## Introduction

Sexual function and its subsequent satisfaction are among the most important aspects of women’s life (1). Cathrine Ingram Fogel considers sexual issues as a major, natural aspect of people’s lives and believes they are beyond just a sexual behavior (2). In fact, sexual issues are one of the priorities of marital life and compatibility in sexual relationships and balance in the couples’ sexual desire are among the major causes of happiness and success in marital life (3). 

WHO defines sexual health as the integration and coordination among mind, emotions, and body, which direct the individuals’ social as well as logical dimensions toward improving their personalities and, at the same time, leads to make relationships and love. Therefore, any disorder resulting from imbalance due to dissatisfaction from sexual relationships may be accompanied by sexual dysfunction (4). Of course, it should be noted that sexual behaviors and desires result from the complex interaction of several factors (5). 

Pregnancy is a condition which leads to organic as well as psychological changes in women and, consequently, can be considered as a major factor in creation and intensification of sexual disorders. Moreover, men’s sexual responses are affected during pregnancy, which may deteriorate the women’s sexual disorders and also create or intensify the sexual disorders in men. The studies conducted on the issue show that in comparison to the pre-pregnancy period, the tendency toward sexual relationships decreases and the rate of sexual disorders highly increases during pregnancy and even several months after the delivery (6, 7). 

Furthermore, some studies have shown sexual deviations, masturbation, oral sex, anal intercourse, and having sexual relationships with other partners (28%) as ways chosen by the men in order to satisfy their sexual needs during their wives’ pregnancy periods (8, 9). Nevertheless, due to the cultural and religious limitations in Iran, a limited number of studies have been conducted on this issue. Therefore, the present study aims to identify sexual disorders during the pregnancy period. 

According to the importance of this problem, not enough research and controversy about this subject, this study is aimed to evaluate sexual problems during pregnancy. We hope the result of this study can increase the knowledge of people, health care providers, psychologists and counselor and improve family stability.

## Materials and methods

The present cross-sectional study was conducted on 257 healthy pregnant women who had referred to Peymanieh Clinic, Jahrom, Iran from April to October 2011.sampling method was convenient and available. A questionnaire containing demographic characteristics was used in order to gather the study data and the information regarding the female sexual function was obtained through interviews and tabulated. 

The questionnaire consisted of two parts, the first of which included the demographic information, such as occupation, age, level of education, place of living, marital age, duration of marriage, and age difference between the couples, while the second part evaluated the participants’ attitudes regarding sexual relationships. After obtaining written informed consents, the questionnaires were completed by two midwifery specialists for the women who had referred to the clinics for pregnancy care services. 

Inclusion criteria were as follows: low-risk pregnancy, absence of abortion, active sexual life defined as sexual activity with penetration within the previous 4 weeks of the evaluation date, and receipt of written informed consent to participate in the study. Exclusion criteria were, chronic diseases (hypertension, heart disease, diabetes), preterm lubor pain, and vaginal bleeding. It should be noted that at the beginning of the study, both the subject and the objectives of the research were explained to the participants and they were reassured about the secrecy of their information. 

Then, written informed consents were obtained, the questionnaires were completed, and the data were extracted. The study was approved by the Ethics Committee of the Jahrom University of Medical Sciences. Female Sexual Function Index (FSFI) questionnaire by Rosen *et al,* consists of 19 questions investigating the subjects in 6 domains of sexual desire, sexual arousal, lubrication, orgasm, sexual satisfaction, and pain during intercourse (10). 

It should be mentioned that sexual desire is covered by questions 1 and 2, excitement by the sum of questions 2, 4, 5, and 6, lubrication by adding questions 7, 8, 9, and 10, orgasm by the sum of questions 11, 12, and 13, sexual satisfaction by adding questions 14, 15, and 16, and pain by summing up questions 17, 18, and 19. In addition, the sexual function total score is ranged from 2-36. Overall, FSFI questionnaire is a general standard one whose reliability and validity were determined by Rosen *et al* in a study conducted in 2000. 

Mohammadi *et al* also performed a study in Shahed University, Iran in 2004 and confirmed the reliability as well as the validity of the questionnaire (11). In the present study, the cut-off point of 26.5 was used for determining the sexual dysfunction; in a way that FSFI <26.5 was considered as suffering from sexual dysfunction and FSFI ≥26.5 was considered as having normal sexual function (12). 

**Statistical analysis**

All the data were analyzed through the SPSS statistical software (Version 11.5) and descriptive statistics were used for demographic variables. Moreover, in order to compute the sexual function total score, ANOVA was first used and since the relationship between the groups was statistically significant, Post Hoc (LSD) was utilized in order to determine the inter-domain correlations in the first, second, and third trimesters. Besides, p<0.05 was considered as statistically significant.

## Results

The present study was conducted on 257 pregnant women in the age range of 18-40 and the mean age of 26.45±4.49 years. In addition, 22.2%, 30.7%, and 47.1% of the study subjects were in the first, second, and third trimesters, respectively. Most of the participants were housewives (29.4%), from urban areas (65.2%), and had high school education (41.6%). Considering the productivity characteristics, 55.6% of the participants were experiencing pregnancy for the first time, 59.5% had no children, and 87.5% had planned pregnancies (Table I). 

According to the results, the participants’ sexual function total score was reported as 19.91±22.45. Furthermore, among the 257 study subjects, 79.1% were suffering from sexual dysfunction (FSFI<26.5), while 52 individuals had normal sexual function (FSFI≥26.5). Moreover, the highest sexual dysfunction was reported in the third trimester (46.2%) and it was reported as 30.5% and 23.4% in the first and the second trimesters, respectively.

As the results showed, the female sexual function score in first and third trimester were lower than second trimester. In addition, a statistically significant relationship was observed among the domains of sexual function in all the 3 trimesters (p<0.05). In fact, the 3 trimesters were significantly correlated with sexual desire (p=0.001), sexual arousal (p=0.004), lubrication (p=0.002), orgasm (p=0.004), and sexual satisfaction (p=0.002). On the other hand, although the mean of pain during intercourse had decreased with the increase in the gestational age, no significant difference was observed among the 3 trimesters regarding this domain (Table II).

Comparison of the domains of sexual function during pregnancy showed the lowest mean to be related to sexual desire (2.29±1.67), sexual arousal (2.39±1.86), orgasm (2.57+2.06), pain during intercourse (2.90±2.28), lubrication (3±2.22), and sexual satisfaction (3.35±2.30). 

Furthermore, 25 women (9.8%) stated that they did not have sexual desires during pregnancy and 18 cases (7%) said that they did not have lubrication. In addition, 29 subjects (11.3%) mentioned that they had not reached orgasm and 17 cases (6.7%) believed that they had not reached the sexual excitement and arousal phase. Besides, 53 women (20.6%) reported no pain during intercourse and only 3 subjects stated that they had no sexual satisfaction during pregnancy. 

Furthermore, a significant correlation was found among all the domains of sexual function (r=0.403-0.927). Also, the most significant correlations were observed between sexual arousal and sexual satisfaction (r=0.927) as well as sexual desire and sexual arousal (r=0.909). Regarding the women’s attitude toward having sexual relationships during pregnancy, 46.3% of the subjects believed that intercourse during pregnancy might damage the fetus and 51.8% considered it to lead to preterm births. 

Also, 52.9% of the study subjects believed intercourse during pregnancy to lead to abortion and 52.9% considered it as the reason for fetal infections. Besides, 24.6% of the participants regarded intercourse during pregnancy as a sin. Finally, 61.9% of the women believed that their sexual attractiveness had decreased during pregnancy. 

**Table I:** Demographic characteristics of the participants (n=257)

**Characteristics **		**Mean ± SD (years)**
Age		26.45 ± 4.49
Husband’s age		31.01 ± 5.49
Marital age		20.51 ± 4.49
Duration of marriage		5.62 ± 5.12
Age difference		5.27 ± 3.18
		**N (%)**
Parity		
	0	153 (59.5%)
	1-3	93 (36.3%)
	>3	9 (3.5%)
Gestational Age (years)		
	<14	57 (22.2%)
	14-28	79 (30.7%)
	>28	121 (47.1%)
Education		
	None	4 (1.6%)
	Primary school	92 (35.5%)
	High school	107 (41.6%)
	University	54 (21%)
Occupation		
	Employed	235 (91.4%)
	housewife	22 (8.6%)

**Table II:** Sexual function score in each domain and sexual function total score in the 3 trimesters

**Domain**	**1** ^st^ ** trimester**	**2** ^nd^ ** trimester**	**3** ^rd^ ** trimester**	**p-value** *				**Total**
	**GA <14 (n=57)** **(Mean ± SD)**	**GA=14-28 (n=79)** **(Mean ± SD)**	**GA >28 (n=121)** **(Mean ± SD)**	**p** **	p (1*2)	p (2*3)	p (1*3)	**n=257**
Desire	3.02 ± 1.27	2.52 ± 1.68	1.58 ± 1.66	0.001	0.28	0.013	0.001	2.29 ± 1.67
Arousal	3.10 ± 1.37	2.69 ± 1.91	1.56 ± 1.90	0.004	0.34	0.021	0.002	2.39 ± 1.86
Lubrication	3.73 ± 1.63	3.34 ± 2.20	2.20 ± 2.39	0.002	0.54	0.007	0.002	3.00 ± 2.22
Orgasm	3.03 ± 1.69	3.07 ± 2.24	1.85 ± 2.06	0.004	0.72	0.007	0.005	2.57 ± 2.06
Satisfaction	4.71 ± 1.63	3.70 ± 2.27	2.51 ± 2.48	0.002	0.41	0.012	0.002	3.35 ± 2.30
Pain	3.39 ± 1.71	3.18 ± 2.25	2.31 ± 2.57	0.313	0.90	0.16	0.026	2.90 ± 2.28
Total score	20.34 ± 8.33	21.42 ± 18.56	16.67 ± 25.26	0.55	0.36	0.33	0.90	19.91 ± 22.45

* p-value post Hoc (LSD) test: between the first and the second trimester, the first and the third trimester, and the second and the third trimester.

** p-value ANOVA test: between first, second, and third trimesters.

## Discussion

Due to the physical as well as psychological changes, sexual relationships tend to change during pregnancy. Moreover, the individuals’ sexual behaviors and attitudes in pregnancy are affected by cultural values, tradition, religious beliefs, physical changes, and obligatory medical limitations. The results of the present study showed that sexual dysfunction increased with the progress of pregnancy; in a way that the highest sexual dysfunction was detected during the 3^rd^ trimester. Moreover, the sexual function score had decreased in the 3^rd^ trimester compared to the 1^st^ and the 2^nd^ trimesters, which is in consistent with the results obtained by Erol *et al* (13). 

The findings of a meta-analysis also revealed that the disorders in orgasm and dissatisfaction from reaching orgasm were more prevalent during the 3^rd^ trimester (14). In fact, the worries about the delivery as well as fetus health and inconvenience with the enlargement of the abdomen are intensified during the 3^rd^ trimester. Therefore, the sexual relationships of a great number of couples are decreased or even cut during this period, which, consequently, leads to disorders in their sexual as well as marital relationships.

Based on the findings of the present study, the sexual function score in the 2^nd^ trimester was higher than those of the 1^st^ and the 3^rd^ trimesters. In fact, due to the physiological changes occurring in the 2^nd^ trimester, most women feel more comfortable with having sexual relationships during this period. According to Kolman, women have more sexual thoughts, experience sex dreams, and begin their sexual behaviors during the 2^nd^ trimester (15). 

Furthermore, vascular congestion of sex organs during the excitement phase tends to be more intense during the 1^st^ and the 2^nd^ trimesters. Therefore, women experience more intense orgasms and even multiple orgasms during the 2^nd^ trimester, which is due to the increase in total serum androgen as well as pelvic congestion and the women’s feeling more comfortable in this period (2). In other studies, also, the women’s sexual function has been reported to increase in the 2^nd^ trimester as well as the beginning of the 3^rd^ trimester, which might be due to the decrease of the couples’ stress about abortion during this period (4).

In the present study, the problem of sexual desire had increased with the progress of pregnancy and a statistically significant difference was observed among the 3 trimesters. Moreover, among the sexual function domains, the lowest mean score was related to sexual desire, which is in line with the results of the study conducted by Rahimi *et al* in Tabriz, Iran (16). Furthermore, the decreasing trend of sexual desire was consistent with the results of the study conducted by Aslan *et al* in Turkey and the one performed by Fok *et al* in China; however, it was in contrast with the findings of the studies conducted by Masters and Johnson, and Angles (17-19). 

Judicibus and Von also believed the sexual desire to reduce in pregnant women, which is more obvious during the 1^st^ and the 3^rd^ trimesters (20, 21). In fact, with the progress of pregnancy, sexual desire, number of intercourses, number of orgasms, and sexual satisfaction decrease in most women. Moreover, fatigue, nausea, vomiting, painful intercourses, and changes in women’s mental images can be considered as the reasons for decrease of women’s sexual desire during the 1^st^ trimester (22). In addition, the reduction of sexual desire in the present research can be justified by considering the fact that most pregnant women are highly concerned about their child during the 3^rd^ trimester and, as a result, they do not pay much attention to sexual relationships in this period (20).

The findings of the present study showed that sexual excitement and arousal had decreased with the progress of pregnancy and a significant difference was observed among the 3^rd^ trimesters (p=0.004), which is in line with the results obtained by Aslan (17). In general, the mother’s imagination of herself as well as her body and her health status affect her sexual desire to a great extent. Nevertheless, the mother’s stress of being seen by the fetus, especially after the beginning of the fetal movements, can lead to a decrease in her sexual excitement during pregnancy (4).

Similar to other domains, lubrication had also decreased in the present study and this difference was statistically significant. In general, vaginal tissues lead to congestion during pregnancy, which results from the increase of the blood vessels as well as circulation in this period. Therefore, pregnant women are in a physiological arousal condition which can lead to either vaginal wetness or vaginal dryness and discomfort (4). 

Moreover, decrease in lubrication results in decrease in sexual arousal. The results of the present study also revealed a positive correlation between lubrication and sexual satisfaction; in a way that as lubrication decreased, sexual satisfaction decreased, as well. The results of the present study revealed a statistically significant difference between the three trimesters regarding orgasm; in a way that as the gestational age increased, orgasm decreased in the study subjects. This finding is consistent with the results obtained by Aslan *et al*, Gokyildiz and Beji, and Nourizadeh *et al* on the reduction of women’s sexual satisfaction with the progress of pregnancy (17). 

In general, enlargement of the abdomen and worrying about hurting the fetus lead to the decrease in sexual satisfaction. In the present study, 11.3% of the women stated that they had not reached orgasm during pregnancy. As pregnancy progressed, sexual satisfaction decreased in the present study. This might be due to the feeling of physical ugliness or not being attractive for the husband and changes in the women’s mental images (22, 23). 

On the other hand, the findings of the present study revealed a significant relationship between sexual satisfaction and being satisfied with pregnancy, feeling of being attractive, and the ability to reach orgasm. Moreover, since there is no need to use the contraceptives during pregnancy, a large number of women are more comfortable with having sexual relationships and reach higher levels of sexual satisfaction during this period.

In the present study, no significant relationship was found between painful intercourses and the progress of pregnancy. Nevertheless, several studies have shown pregnancy to have negative effects on orgasm, sexual satisfaction, and painful intercourses. Overall, painful intercourses during pregnancy might be due to the anatomical changes of this period as well as hypertrophy of pelvic viscera (24-27). 

Regarding the women’s attitude toward having sexual relationships during pregnancy, the findings of the present study showed that more than half of the study subjects believed intercourse during pregnancy to result in damaging the fetus, preterm births, abortion, and fetal infections. In line with the findings of the present study, the results of the studies conducted by Heidari *et al* in Tehran, Rahimi *et al* in Tabriz, and Pasha *et al* in Babol, Iran also showed worries about hurting the fetus, abortion, preterm births, and fetal infections in more than half of their study subjects (16, 28, 29). Moreover, the findings of the study conducted in Taiwan showed that 80% of the women were worried about hurting the fetus during sexual relationships in pregnancy, which is quite considerable compared to the results of the present study. Investigations performed in Canada and Karachi, Pakistan and the study conducted by Orji *et al* have also confirmed the worries about hurting the fetus as one of the reasons for the decrease in sexual relationships during pregnancy (30-33). 

Therefore, Orji *et al* believe that the health authorities must make attempts in order to create positive attitudes in the couples regarding having sexual relationships during pregnancy (33). Furthermore, several studies have shown no significant relationships between intercourse during pregnancy and abortion, premature rupture of the membranes, and preterm births (34, 35).

About a quarter of women believe that sexual activity during pregnancy is a sin. It seems that strict religious manners and women feeling about the fetus as a third person in sexual activity, make the sense of being guilty and induce some problems in sexual activity. There was a significant relation between sexual activity and feeling guilty in the Bayrami *et al* study in Tabriz, Iran. They showed that most sexual dysfunction is seen in the pregnant woman, believed their fetus injured during sexual activity (36). 

Nicols mentioned if sexual activity continued by partners agreement, no problem is occurred. Feeling of being guilty, shame and sexual rejection in one of the partners result in distress and sexual dysfunction (37). In the present study, no association was observed between the FSFI and variables such as educational level, maternal age, occupation, or parity. Although 92% of the participants had primary school educational level. As also reported in previous studies that evaluate in sexual function during pregnancy, no association was detected here between FSFI score and educational level by Haines *et al*, and Pauls *et al* (38, 39).

Regarding maternal age, the present results confirm previous investigations that did not detect an association between this variable and sexual function in pregnant women by Haines *et al*, and Pauls *et al* (38, 39). No association between parity and sexual function was observed in the present study. This is in agreement with previous reports by Gruszecki *et al*, and Pauls *et al* (39, 40). Sydow showed that there is no relation between sexual variable and demographic charactristics such as education, nationality, economic status, duration of marriage, job, social class and job satisfaction during pregnancy and after delivery (14). 

## Conclusion

The results of the present study revealed considerable sexual dysfunction in pregnant women (79.1%). Wrong beliefs about sexual relationships during pregnancy play a major role in the incidence of sexual dysfunction among the couples and, at the same time, can negatively affect the marital relationships. Therefore, it seems quite necessary to hold marital counseling classes in health centers in order for the women to discuss their sexual problems and benefit from the counseling methods. These consultations can also play a key role in improving the women’s reproductive health. 

Since sexual relationships are among the most private issues of marital life and individuals may not be able to frankly talk about their sexual issues due to the cultural and religious limitations of our society, some study subjects might not have been quite honest while providing information regarding their sexual issues. 
